# Progress Toward Poliomyelitis Eradication — Afghanistan, January 2016–June 2017

**DOI:** 10.15585/mmwr.mm6632a5

**Published:** 2017-08-18

**Authors:** Maureen Martinez, Hemant Shukla, Joanna Nikulin, Mufti Zubair Wadood, Stephen Hadler, Chukwuma Mbaeyi, Rudolph Tangermann, Jaume Jorba, Derek Ehrhardt

**Affiliations:** ^1^Global Immunization Division, Center for Global Health, CDC; ^2^Polio Eradication Department, World Health Organization, Geneva, Switzerland; ^3^Division of Bacterial Diseases, National Center for Immunization and Respiratory Diseases, CDC; ^4^Division of Viral Diseases, National Center for Immunization and Respiratory Diseases, CDC.

Afghanistan, Pakistan, and Nigeria remain the only countries where the transmission of endemic wild poliovirus type 1 (WPV1) continues (*1*). This report describes polio eradication activities, progress, and challenges in Afghanistan during January 2016–June 2017 and updates previous reports (*2*,*3*). Thirteen WPV1 cases were confirmed in Afghanistan in 2016, a decrease of seven from the 20 cases reported in 2015. From January to June 2017, five WPV1 cases were reported, compared with six during the same period in 2016. The number of affected districts declined from 23 (including WPV1-positive acute flaccid paralysis [AFP] cases and positive environmental sewage samples) in 2015 to six in 2016. To achieve WPV1 eradication, it is important that Afghanistan’s polio program continue to collaborate with that of neighboring Pakistan to track and vaccinate groups of high-risk mobile populations and strengthen efforts to reach children in security-compromised areas.

## Immunization Activities

Estimated routine immunization coverage of infants with 3 doses of oral poliovirus vaccine (OPV3) in Afghanistan was 60% in both 2015 and 2016 (*4*). The percentage of children aged 6–23 months with nonpolio acute flaccid paralysis (NPAFP) who received 3 doses of OPV through routine immunization programs is used as a proxy indicator for OPV3 coverage nationally and was 65% in 2015 and 67% in 2016. However, there was wide regional variation, ranging from 100% in the Central provinces to 28% in the Southern province of Helmand. The proportion of children aged 6–23 months with NPAFP who had never received OPV through routine immunization or supplementary immunization activities (SIAs)* (i.e., “zero-dose” children) was approximately 1% nationally in 2016, virtually unchanged from 2015. The highest percentages of zero-dose children were reported from four provinces in 2016: Paktika (17%), Badghis (7%), Helmand (6%), and Nangarhar (2%).

During January 2016–May 2017, SIAs targeted children aged <5 years for receipt of one or both of the following vaccines: bivalent OPV (types 1 and 3) or trivalent OPV (types 1, 2, and 3 [until the global withdrawal of all type 2-containing OPVs, including tOPV, on May 1, 2016]). In addition, inactivated polio vaccine (IPV) was administered during SIAs to children aged 4–59 months who had not received IPV during a previous campaign and who lived in the 47 districts designated to be at very high risk for poliovirus transmission or in areas that had been inaccessible for three or more previous SIAs. During this period, 15 SIAs were conducted using OPV with or without IPV, including six national immunization days (NIDs) and nine subnational immunization days (sNIDs). Five case-response vaccination campaigns (i.e., mop-up campaigns) and five short-interval additional dose rounds (SIADs)^†^ were also held.

Vaccination of children aged <10 years continued at border crossing points with Pakistan and throughout the country along major travel routes and at the entry and exit points to and from inaccessible areas. Teams of vaccinators at these locations reached approximately 11 million children with OPV in 2016 and approximately 5 million during January–May 2017.

Insecurity associated with active conflicts limits the program’s ability to reach all children with polio vaccine during SIAs. The polio program addresses issues of access by deferring the campaign in areas with active fighting, and by engaging in dialogue with local influencers, which, to date, has demonstrated limited success in gaining access for vaccination activities.

During the March 2016 NIDs, among 9,523,420 children aged <5 years targeted for vaccination, 184,363 children (1.9%) were missed because of inaccessibility, including 11,684 (0.1%) in the Southern provinces of Helmand, Kandahar, and Uruzgan; 25,869 (0.3%) in the Eastern provinces of Kunar, Nangarhar, and Nuristan; and 146,810 (1.5%) in the Northeastern province of Kunduz. During the October 2016 NIDs, the number of inaccessible children increased to 393,737, representing 4.4% of those targeted. The number of inaccessible children was reduced during the March 2017 NIDs to 98,915 (1%) and further to 80,899 during the May 2017 NIDs. The reductions are reflective of the recent progress in the Kunduz province in Northeast, where, after nearly 1.5 years of campaign bans, full access was obtained.

Despite the challenges of inaccessibility, the largest numbers of children missed during campaigns live in accessible areas of Afghanistan. Postcampaign evaluation surveys reveal that children are missed in these areas because of reasons that include the child was not at home; vaccine was refused; or the child was sick, sleeping, or a newborn. The percentages of children missed during campaigns in accessible areas ranged from 5.7% in the March 2016 NID to 4.1% in the March 2017 NID. The Southern part of the country continues to record the largest percentages of children in accessible areas missed, with 9.5% missed in March 2016 and 5.8% in March 2017.

Lot Quality Assurance Sampling^§^ surveys are used to assess the quality of SIAs. Campaigns in 2016 showed a nearly consistent monthly reduction in the number of failed lots (rejected at 80% threshold) from 25% rejected in the March 2016 NID down to 9% in the May 2017 NID, indicating an improvement in SIA quality.

## Poliovirus Surveillance

**AFP surveillance.** Afghanistan has an extensive AFP surveillance network including reporting sites at government and private health facilities, shrines, and by traditional healers. This network is supplemented by an extensive network of reporting volunteers that has increased 66%, from 17,218 in 2015 to 28,543 in 2017. In 2016, the annual national NPAFP rate was 14.4 per 100,000 children aged <15 years (range = 9.8–20.4 per 100,000; surveillance target = 2/100,000 children aged <15 years) (Table). The percentage of AFP cases with adequate stool specimens^¶^ collected was 92.2% (range = 85.2%–98.2%; target = 80%). No polio-compatible AFP cases were reported during the period covered by this report. Analysis of surveillance data shows comparable sensitivity across different access categories. The NPAFP rate exceeded 10 per 100,000 children aged <15 years, and the percentage of AFP cases with adequate stool specimens exceeded 85% across areas with varying levels of inaccessibility related to security challenges.

**TABLE:** Acute flaccid paralysis (AFP) surveillance indicators and reported cases of wild poliovirus (WPV), by region and period — Afghanistan, January 2016–June 2017*

Region of Afghanistan	AFP surveillance indicators (2016)			No. WPV cases reported		
	No. AFP cases	Rate of nonpolio AFP^†^	% of AFP cases with adequate specimens^§^	January–June 2016	July–December 2016	January–June 2017
**All regions**	**2,891**	**14.4**	**92.2**	**6**	**7**	**5**
Badakhshan	56	9.8	98.2	0	0	0
Northeastern	285	12.6	92.2	0	0	1
Northern	319	12.7	90.9	0	0	0
Central	507	11	96.4	0	0	0
Eastern	405	20.4	95.4	4	0	0
Southeastern	261	13.2	91.4	0	7	0
Southern	588	16.7	85.2	2	0	4
Western	470	17.6	94.0	0	0	0

* Data current through June 30, 2017.

^†^ Per 100,000 children aged <15 years. Surveillance target is 2/100,000 children aged <15 years.

^§^ Surveillance target is that at least 80% of AFP cases have “adequate” stool specimens collected. “Adequate” stool specimens are defined as two stool specimens of sufficient quality for laboratory analysis, collected ≥24 hours apart, both within 14 days of paralysis onset, and arriving to a World Health Organization–accredited laboratory by reverse cold chain and with proper documentation.

**Environmental surveillance.** Since September 2013, Afghanistan has been conducting supplemental poliovirus surveillance through sampling of sewage at designated sites. There are currently 17 active sites in six provinces throughout the country (Helmand and Kandahar in the Southern region, Kunar and Nangarhar in the Eastern region, Kabul in the Central region, and Khost in the Southeastern region). Three of these sites were added in 2016 (Kandahar and Nangarhar) and 2017 (Khost). During this time, sampling frequency also increased from monthly to biweekly in the Southern region. In 2015, among 148 specimens, 19 (6%) tested positive for WPV1. In 2016, only two (1%) of 184 specimens tested positive for WPV1. As of June 2017, seven (5%) of 150 specimens have tested positive for WPV1. The seven positive samples were collected at sites in Nangarhar (three specimens), Kandahar (three), and Helmand (one).

## Epidemiology of WPV and Vaccine-Derived Poliovirus (VDPV) Cases

**FIGURE 1:**
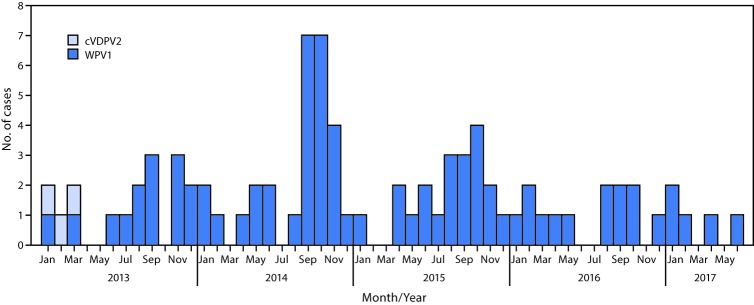
Number of cases of wild poliovirus type 1 (WPV1) and circulating vaccine-derived poliovirus type 2 (cVDPV2), by month and year of paralysis onset — Afghanistan, 2013–2017

**FIGURE 2:**
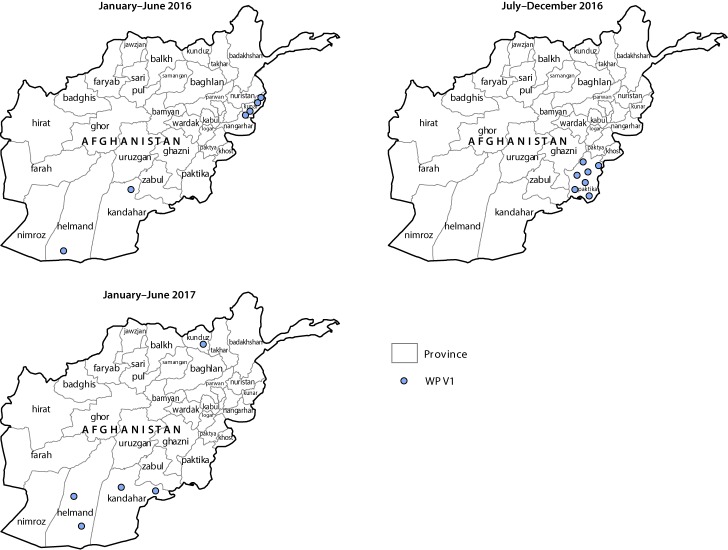
Cases of wild poliovirus type 1 (WPV1), by province — Afghanistan, January 2016–June 2017* * Each dot represents one case. Dots are randomly placed within provinces.

During 2016, 13 WPV1 cases were confirmed in Afghanistan, compared with 20 in 2015. Five cases were confirmed during January–June 2017, compared with six during the same period in 2016 (Figure 1) (Figure 2). Since 2014, when 28 cases were detected in Afghanistan, the number of WPV cases has declined each year. In 2016, the number of districts reporting polio cases had declined to four, from 16 in 2015. Seven (54%) of the 13 cases in 2016 were reported from Bermel district in the Southeastern province of Paktika, four (31%) were reported from Shigal wa Sheltan district of the Eastern province of Kunar, and one each (8%) was detected in the Southern provinces of Helmand (Nawzad district) and Kandahar (Shahwalikot district). As of June 2017, five cases have been reported, one from Kunduz province and two each from Helmand and Kandahar provinces. Among all 18 cases reported from January 2016 to June 2017, 13 were in children aged <36 months, seven of whom had never received OPV; one child had received 1 dose, two had received 2 doses, one had received 4 doses, and two had received ≥5 doses. Among the 18 patients, 16 had never received OPV through the routine immunization program. 

Genomic sequence analysis of poliovirus cases and environmental isolates revealed multiple episodes of cross-border transmission in 2016–2017 from Pakistan to Afghanistan, with some sustained local transmission in Afghanistan. Among the seven positive environmental isolates identified from January 2016 to June 2017, five show closest genetic ties to Pakistan strains, with two indicating local circulation in Kandahar and Nangarhar. No cases of WPV3 or circulating vaccine-derived poliovirus type 2 (cVDPV2)** have been detected in Afghanistan since April 2010 and March 2013, respectively (Figure 1). In December of 2016, one ambiguous vaccine-derived poliovirus (aVDPV)^††^ was detected in Bermel district of Paktika province.

## Discussion

During the period covered by this report, the geographic scope and genetic diversity of WPV1 detected in Afghanistan has continued to decline from previous years. The polio program in Afghanistan established a national emergency operations center (EOC) in 2015 and regional EOCs in 2016 to ensure that all regions of the country are monitored and receiving local support for polio activities. The 2016–2017 National Emergency Action Plan for polio includes a strong focus on management, accountability, and enhanced data quality along with interventions for reaching unreached children in both accessible and inaccessible areas. In addition to tracking mobile populations, the country has increased coordination with other international agencies such as the International Organization for Migration and the United Nations High Commissioner for Refugees and separated the high-risk mobile populations into four groups: 1) straddlers (persons who travel regularly between the border areas of Afghanistan and Pakistan), 2) long distance travelers, 3) nomads, and 4) returnees, with a uniquely targeted strategy for tracking each group’s movement and vaccinating their children.

To improve the coordination between countries, the EOCs of Afghanistan and Pakistan established cross border focal points in 2016. Since then, the synchronization of campaigns, regular meetings, data sharing, and collaboration on case investigations and responses, cross-border vaccinations, and mobile population tracking have improved the ability to vaccinate children moving between the two countries.

Although there have been noted improvements in SIAs, suboptimal campaign quality in Southern Afghanistan continues to be a challenge. The polio program has identified 47 districts where OPV-IPV SIAs have been conducted that are at very high risk for polio transmission. Microplans for polio SIAs are being revised to prioritize high-risk districts. Campaigns have been extended to include activities to better identify and vaccinate missed children. After 3 days of vaccination, teams now spend the fourth day reviewing data on missed children and planning targeted strategies to reach them on the fifth day. Independent campaign monitoring has been incorporated, particularly for areas with security challenges. Religious leaders are being engaged from the national to the community level to participate in social mobilization efforts.

Transit teams have been established in a more targeted manner at the entry and exit points into inaccessible areas and along travel routes. Cross-border teams are located at all formal and informal border crossing points. The use of community members as Immunization Communications Network volunteers has accelerated and is contributing to increases in acceptance of vaccination by families who had earlier refused and in catching up missed children between campaigns. The Immunization Communications Network is also proving helpful in identifying high-risk mobile population groups and their movement patterns.

To accomplish eradication, it is essential that the polio program in Afghanistan continue to refine its strategies for vaccinating remaining pockets of missed children and reaching the high-risk mobile population. The polio program will benefit from completing its commitment to dedicate staff members’ time to supporting routine immunization without compromising core polio-eradication activities. Detection of orphan viruses, which are >1% divergent from the most closely related isolate, indicating extended undetected circulation of poliovirus, along with continued close genetic linkages with Pakistan viruses, highlight the need for Afghanistan and Pakistan to continue to prioritize coordination to improve surveillance, and to track and vaccinate their mobile populations, thereby stopping the ongoing cross-border transmission and reducing the risk for poliovirus circulation in hard-to-reach areas of Afghanistan.

**Summary**

**What is already known about this topic?**

Afghanistan is one of three countries where transmission of indigenous wild poliovirus (WPV) has never been interrupted. The Southern and Eastern regions of the country continue to be the main areas where WPV cases and positive environmental samples are identified.

**What is added by this report?**

The number of WPV type 1 cases reported in Afghanistan has declined yearly since 2014 when 28 cases were reported to 13 in 2016, indicating continued progress toward eradication. Factors contributing to this decline include increased focus on hard-to-reach populations, improved partner coordination, and successful negotiation to obtain access for campaigns, resulting in fewer children being missed during campaigns. During the October 2016 National Immunization Days (NIDs) 4.4% of children were missed because of security issues; <1% of children were missed because of insecurity during the May 2017 NIDs. The identification of a new corridor for transmission between Afghanistan and Pakistan in the Southeastern region, as well as ongoing case detection in the Southern region, highlight persistent immunity gaps.

**What are the implications for public health practice?**

To interrupt poliovirus transmission, Afghanistan’s polio program will benefit from further refinement of strategies to vaccinate hard-to-reach populations and improve campaign quality, especially in the south. Prioritizing coordination between Afghanistan and Pakistan on surveillance and vaccination activities for their shared mobile populations is important to stop ongoing cross-border transmission and reduce the risk for poliovirus circulation in hard-to-reach areas of Afghanistan.

## Footnotes

* Mass vaccination campaigns that aim to administer additional doses of OPV to each child aged <5 years, regardless of vaccination history.

† Short-interval, additional dose campaigns are used for case-response vaccination after detection of a WPV case or during negotiated periods of nonviolence in otherwise inaccessible areas, to provide 2 doses of monovalent or bivalent OPV within 1–2 weeks.

§ Lot Quality Assurance Sampling is a rapid survey method used to assess the quality of vaccination activities after SIAs in predefined areas, such as health districts (referred to as “lots”), using a small sample size. Lot quality assurance sampling involves dividing the population into “lots” and randomly selecting persons in each lot. If the number of unvaccinated persons in the sample exceeds a predetermined value, then the lot is classified as having an unsatisfactory level of vaccination coverage, and mop-up activities are recommended. If the threshold of ≥80% is met, the area/district is classified as having “passed,” although mop-up activities might still be indicated in certain areas.

¶ The indicator for stool adequacy requires that at least 80% of AFP cases should have “adequate” stool specimens collected. “Adequate” stool specimens are defined as two stool specimens of sufficient quantity for laboratory analysis, collected at least 24 hours apart, within 14 days after the onset of paralysis, and arriving in the laboratory by reverse cold chain and with proper documentation.

** In severely underimmunized populations, high levels of susceptibility in children enable prolonged circulation of excreted vaccine-derived polioviruses in the community. Vaccine viruses that circulate uninterrupted for long periods can mutate and, over the course of 12–18 months, reacquire neurovirulence and can cause paralysis. These viruses are known as circulating vaccine-derived polioviruses (cVDPVs).

†† aVDPVs are vaccine-derived polioviruses that are either isolated from persons with no known immunodeficiency, or isolated from sewage and whose ultimate source is unknown.
