# *Erratum*: Vol. 66, No. 32

**DOI:** 10.15585/mmwr.mm6650a6

**Published:** 2017-12-22

In the report “Progress Toward Poliomyelitis Eradication — Afghanistan, January 2016–June 2017,” on page 857, the last sentence of the last paragraph should have read “Detection of orphan viruses, which are **≥1.5%** divergent from the most closely related isolate, indicating extended undetected circulation of poliovirus, along with continued close genetic linkages with Pakistan viruses, highlight the need for Afghanistan and Pakistan to continue to prioritize coordination to improve surveillance, and to track and vaccinate their mobile populations, thereby stopping the ongoing cross border transmission and reducing the risk for poliovirus circulation in hard-to-reach areas of Afghanistan.”

